# Some Work and Some Play: Microscopic and Macroscopic Approaches to Labor and Leisure

**DOI:** 10.1371/journal.pcbi.1003894

**Published:** 2014-12-04

**Authors:** Ritwik K. Niyogi, Peter Shizgal, Peter Dayan

**Affiliations:** 1 Gatsby Computational Neuroscience Unit, University College London, London, United Kingdom; 2 Center for Studies in Behavioral Neurobiology, Concordia University, Montreal, Quebec, Canada; Indiana University, United States of America

## Abstract

Given the option, humans and other animals elect to distribute their time between work and leisure, rather than choosing all of one and none of the other. Traditional accounts of partial allocation have characterised behavior on a macroscopic timescale, reporting and studying the mean times spent in work or leisure. However, averaging over the more microscopic processes that govern choices is known to pose tricky theoretical problems, and also eschews any possibility of direct contact with the neural computations involved. We develop a microscopic framework, formalized as a semi-Markov decision process with possibly stochastic choices, in which subjects approximately maximise their expected returns by making momentary commitments to one or other activity. We show macroscopic utilities that arise from microscopic ones, and demonstrate how facets such as imperfect substitutability can arise in a more straightforward microscopic manner.

## Author Summary

Dividing limited time between work and leisure when both are attractive is a common everyday decision. Rather than doing one exclusively, humans and other animals distribute their time between both. Traditional explanations of this phenomenon have studied the macroscopic average times spent in both. By contrast, we develop a microscopic framework in which we can model the real-time decisions that underpin these averages. In the framework, subjects' choices are approximately optimal, according to a natural, microscopic, utility function. We show that the assumptions of previous theories are not necessary for partial allocation to be optimal, and show possibilities and limits to the integration of macroscopic and microscopic views. Our approach opens new vistas onto the real-time processes underlying cost-benefit decision-making.

## Introduction

When suitably free, humans and other animals divide their limited time between work, i.e., performing employer-defined tasks remunerated by rewards such as money or food, and leisure, i.e., activities pursued for themselves that appear to confer intrinsic benefit. The division of time provides insights into these quantities and their interaction, and has been addressed by both microeconomics and behavioral psychology.

Microeconomic labor supply theorists [1] have adopted a normative perspective, formulating what a rational agent *should* do. Accounts from behavioral psychology have been descriptive, detailing *how* subjects allocate their time, for example, proportionally to the relative payoffs from work and leisure [2]–[8]. Common to these approaches is the coarse, *macroscopic* timescale at which behavior is characterised, focusing on average times spent in work and leisure. By contrast, *microscopic* analyses characterise the fine temporal topography of work and leisure choices, and so offer a foundation for examining, rather than averaging away, rich psychological and neural processes. Tying microscopic and macroscopic choices together is known to be difficult in general [9], because the former involves a much more elaborate state space than the latter.

Here, we build an approximately optimal stochastic control theoretic model of decision-making at a microscopic level. We show how averaging over the microscopic choices yields a characterizable superset of traditional macroscopic theories, and casts the assumptions necessary for the latter to capture partial allocation in a different light. We make the novel prediction that partial allocation requires neither stochastic choices (as generally assumed by accounts from behavioral psychology) nor the marginal utility of leisure to depend on the amount of work performed. We use a simplification of a particularly stark labor task as a paradigm example to show how macroscopic and microscopic theories of the partial allocation of time between work and leisure can be tied. We therefore do not attempt to model actual data from this task; a qualitative account is available in [10].

## Results

### Task and experiment

We consider a Cumulative Handling Time task [11], [12] in which subjects must accumulate work up to a total time-period called the *price* 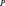 (see Table 1 for a list of symbols and their meanings) to gain a reward. The price and the objective strength of the reward are defined by the experimenter. Note that the price is an experimenter determined time-period, hence we shall use “long” and “short” to denote its duration. Subjects are free to distribute leisure bouts in between work bouts (Fig.S1A). The CHT controls both the (average) minimum inter-reward interval and the amount of work required to earn a reward. This makes the CHT a generalisation of common schedules of reinforcement such as Fixed Ratio, or Variable Interval, which control one but not the other.

**Table 1:** List of symbols.

Symbol	Meaning
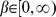	inverse temperature or degree of stochasticity-determinism parameter
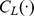	microscopic utility of leisure
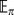	expected value with respect to policy 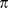
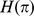	entropy
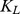	marginal utility of linear microscopic utility of leisure
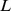	leisure
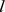	cumulative amount of time spent in leisure
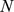	total number of rewards accrued
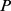	Price
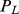	price at which 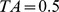 , for a maximum subjective reward intensity 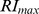
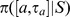	policy or choice rule: probability of choosing action 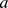 , for duration 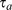 from state 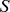
post	post-reward
pre	pre-reward
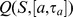	expected return or (differential) *Q*-value of taking action 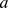 , for duration 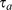 from state 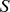
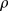	reward rate
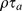	average foregone reward for taking action 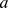 for duration 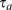
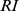	(subjective) Reward Intensity
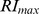	maximum (subjective) Reward Intensity
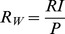	payoff
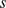	degree of substitutability between rewards (or work) and leisure
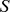	state
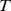	trial duration
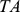	Time Allocation
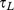	duration of leisure
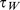	duration of work
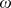	cumulative amount of time spent in work
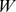	work
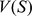	expected return or value of state 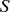
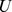	macroscopic utility

Reward and leisure are both assumed to enjoy a subjective worth. We call these *microscopic utilities* to distinguish them from the *macroscopic utilities* used by traditional theories. The microscopic utility of the former is called the (subjective) *reward intensity* (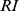, in arbitrary units); the ratio of this to the price is called the payoff (or in economic nomenclature, wage rate) 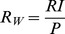. For simplicity, we consider the objective price, recognising that its subjective value may differ. We explore different functional forms for the presumed microscopic utility of leisure.

This paradigm was originally developed in the context of rats pressing down an unweighted lever to gain non-satiating, brain stimulation reward (BSR), or alternatively choosing leisure in the form of resting, grooming, exploring, etc. However, as noted above, we do not model data, but rather consider an abstracted version of the task in order to concentrate on the relationship between microscopic and macroscopic descriptions.

### Macroscopic and microscopic analyses

The key macroscopic statistic is the Time Allocation (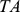): the proportion of trial time that the subject spends working [2]. Fig.S1B shows example *TA*s for a typical subject. As expected, the *TA* increases with reward intensity and decreases with price. A microscopic analysis, as shown by *ethograms* in (Fig.S1C), considers the detailed temporal topography of choice, recording when and for how long each act of work or leisure occurred. Note that at intermediate payoffs, when partial allocation is most noticeable, subjects consume almost all leisure immediately after getting a reward, and then work continuously for each entire price [13].

### Traditional macroscopic accounts: I

#### Microeconomics: Labor supply theory

**Figure 1:**
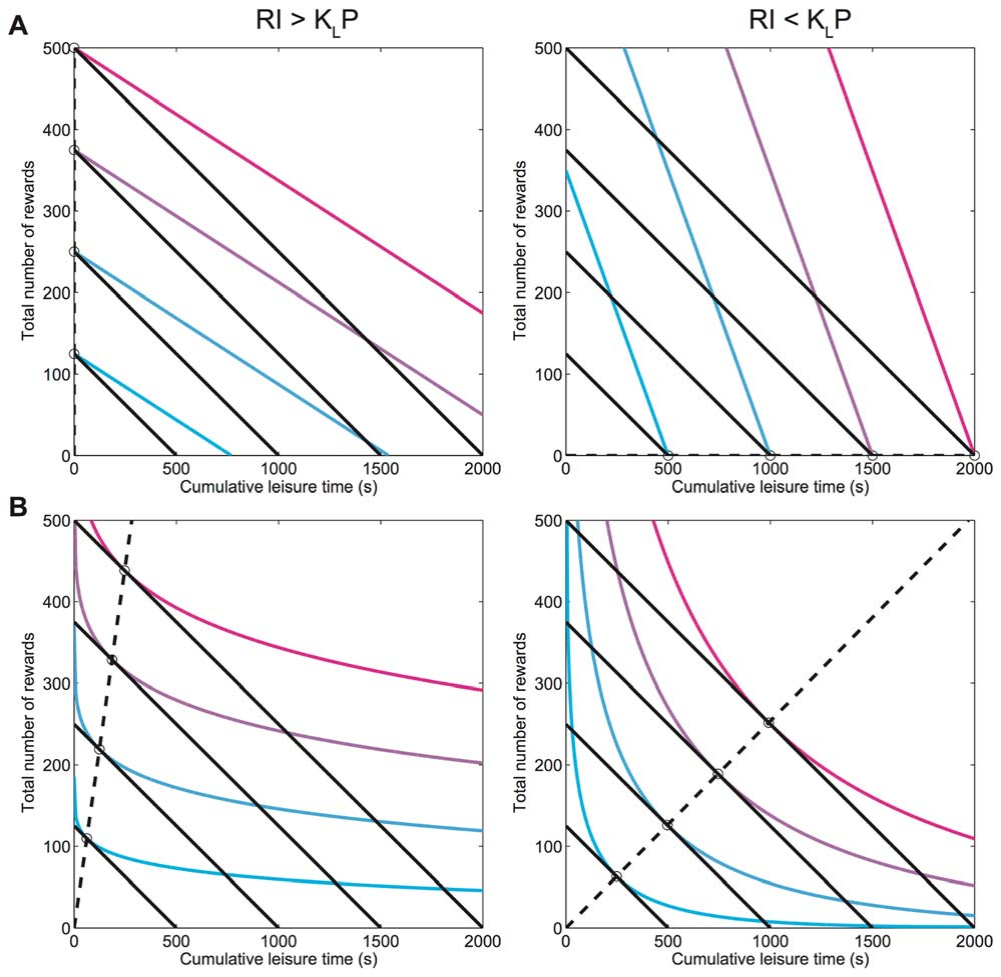
Indifference curves (ICs) of the labor supply theory model in Eq.(1). Left: Returns from work exceed those from leisure (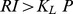) and right: vice versa (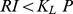). Solid black lines show the budget constraint (BC): trial duration 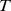 is constant. Open circles show optimal combination of rewards and leisure for which macroscopic utility is maximised subject to BC. Dashed black lines denote the path through theoretically predicted optimal leisure and reward combinations as 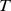 is increased. A) perfect substitutability between rewards (work) and leisure (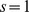). Optimal combination is when the subject works all the time and claims all rewards if 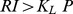, and engage in leisure all the time otherwise. B) imperfect substitutability (e.g. 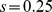). Optimal combination comprises non-zero amounts of work and leisure.

In labor supply theory [1], subjects are assumed to maximize their *macroscopic* utility by trading (i) income from working (worth 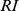 per reward), against (ii) leisure (worth, in the simplest case, a marginal utility of 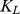 per unit time). Let 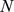 be the *total* number of rewards that a subject accumulates, and 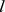 be the *cumulative* amount of time spent in leisure. A commonly assumed form of *macroscopic utility function* is [14], [15]. 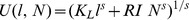
(1)where 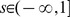 is a dimensionless number representing the degree of *substitutability*, the willingness to replace rewards (or work) with leisure. Fig.1 shows the indifference curves (IC)–contours of equal utility. A subject is indifferent between combinations of these goods along an IC, but combinations on an IC with greater utility are preferred. The slope of an IC, the negative of which is called the *marginal rate of substitution*, shows how willing a subject is to substitute one good with the other, depending on how much of each it has already accumulated. Given a fixed total trial time (a budget constraint; BC Eq. (A-1) in Text S1), subjects must maximise their macroscopic utilities; this occurs for the combination of goods at which the BC is tangent to an IC or is at a boundary.

Work and leisure are perfect substitutes (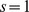 in Eq. (1)) for subjects who are willing to substitute work for leisure at the same rate, irrespective of the amount of either already consumed. The ICs become (negatively sloped) straight lines. The optimum allocation is then at the boundary with all work (if returns from work exceed those from leisure, i.e. 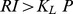) or all leisure (otherwise). This would make *TA* a step-function of the relative returns from work and leisure (black curves in Fig.1A), an outcome that is not observed empirically.

**Figure 2:**
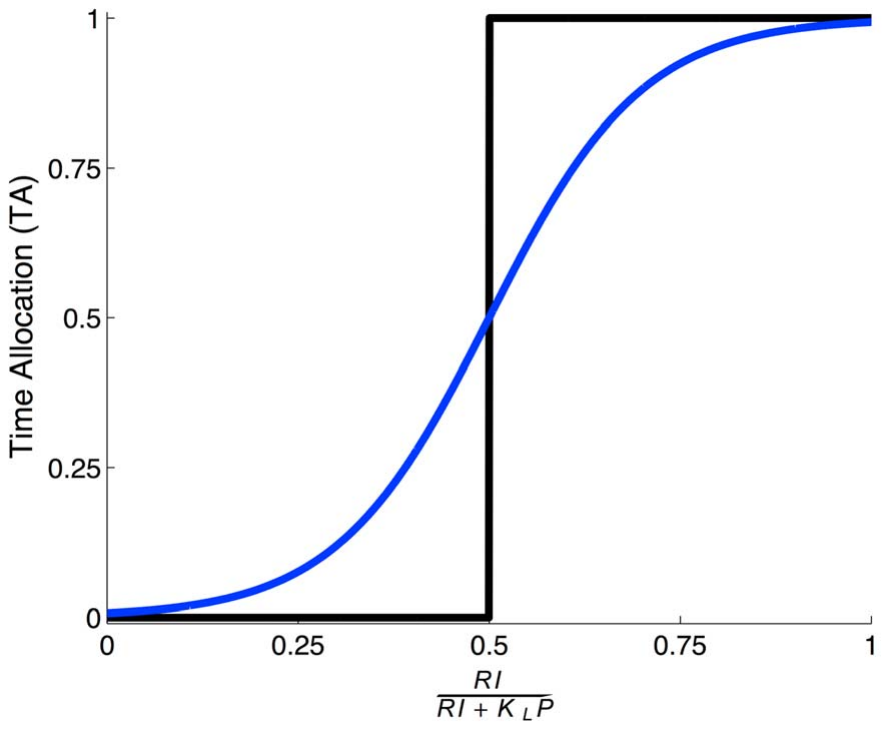
Time allocation from labor supply theory. *TA* as a function of the relative returns from work and leisure predicted by labor supply theory model in Eq. (1). Black and blue curves show the cases of perfect (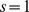) and imperfect substitutability (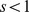), respectively.

However, if work and leisure are imperfect substitutes (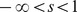 in Eq. (1)), then leisure is preferred more if the subject has worked more, and vice versa even for deterministic subjects. The slope of the IC decreases as additional amounts of leisure are consumed. The optimal combination includes both rewards (work) and leisure, making *TA* a smooth function of the relative returns from work and leisure (blue curves in Fig.2, Eq. (A-2) in Text S1), as is observed empirically.

Of critical psychological importance is the relationship between the macroscopic marginal utility of leisure (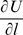) and the amount of work so far done. For imperfect substitutability associated with the utility function of Eq.(1), the former depends on the latter. By contrast, we show in both deterministic and stochastic settings that this is not necessary to achieve partial allocation. The possibilities of non-determinism, which is experimentally ubiquitous, can be treated in various ways, including traditional random utility models [16], [17].

### Normative microscopic approach: Micro SMDP model

**Figure 3:**
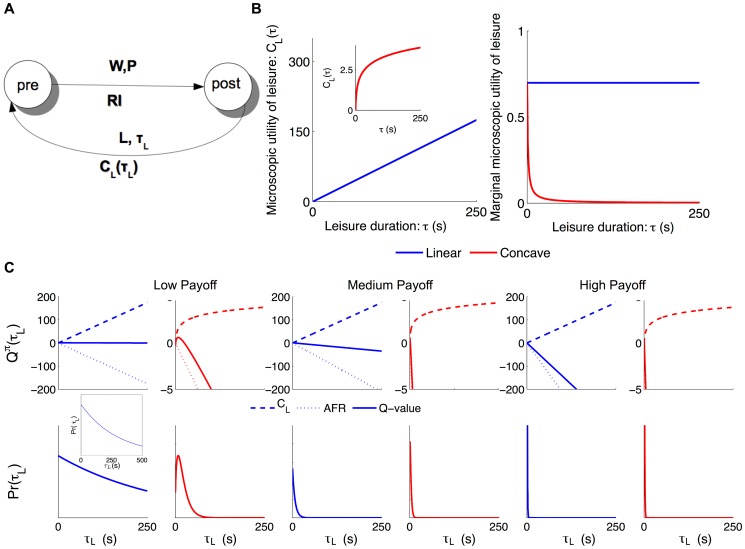
Micro SMDP model, microscopic utilities of leisure and policies. A) The infinite horizon Micro semi-Markov decision process (Micro-SMDP). States are characterised by whether they are pre- or post-reward. Subjects choose not only whether to work or to engage in leisure, but also for how long to do so. For simplicity, we assume that a subject pre-commits to working for the entire price duration when it works. Then it receives a reward of reward intensity 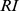 and transitions to the post-reward state. In the post-reward state, by choosing to engage in leisure for a duration 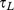, it gains a microscopic benefit of leisure 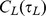 and then returns to pre-reward state; this cycle repeats. B) Left: canonical microscopic utility of leisure functions 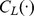, right: the marginal microscopic utility of leisure. For simplicity we considered linear 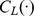 (blue); whose marginal utility is constant and concave (here logarithmic) 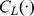 (red) whose marginal utility is always decreasing. C) 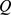-values and policies for engaging in leisure for low, medium and high payoffs. In upper panels, dashed, dotted and solid curves show: 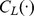, AFR and 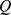-values, respectively.

Labor supply theory and generalized matching average over the temporal topography shown in Fig.S1C). By contrast, we follow [10], [18], [19] in formulating a so-called micro Semi-Markov Decision Process (SMDP) [20], [21] (Fig. 3A) with actions, states, and utilities, for which policies (i.e., the stochastic choices of actions at states) are quantified by the average reward per unit time accrued over the long run. We formulated the general normative, microscopic theoretical framework in [10]. Here we delineate a simplified model pertinent to the partial allocation problem.

#### Actions and states

Subjects choose what action (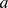) to do, and for how long (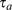). The longer the duration, the more the forgone opportunity to collect rewards for other actions they could instead have been doing during that time. In [10], we developed a fully detailed model of the example CHT task. This model was faithful to the task in allowing the subject to choose the length of each work bout, including distributing leisure inbetween work bouts prior to attaining the price. Here, however, in the interests of an analytical treatment of the partial allocation problem, we model a simplified version of the task in which subjects are *assumed* to work for the entire price. In fact, this is evident in the data (Fig.S1C)), and has been shown to arise from optimization in the face of stochasticity as we showed in [10]. In this simplification, there are just two states: 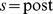
- and 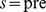-reward. In the former, the subject consumes leisure (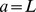) for a freely chosen duration 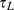; then the state becomes pre-reward. If 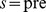, the subject works (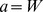) for the entire price 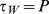, collects a reward and transitions to the post-reward state. The cycle then repeats.

#### Utilities

The *microscopic* utility of the external reward is the subjective reward intensity 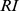. The microscopic utility of leisure 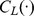 is innate and assumed to depend on its duration, but not any other reward or cost, or the amount of work performed. Based on findings in the case of discrete choices [22]–[24], we expect aspects of these utilities to be discernable through neuroscience experiments; one of our main intents is to construct a framework in which such inferences are precise.

Critically, the assumptions of our microscopic utility function are different from that of the macroscopic utility function, from labor supply theory, in Eq.(1), which assumes that when work and leisure are imperfect substitutes, the macroscopic marginal utility of leisure (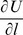) depends on the amount of work performed or the number of rewards received. In particular, we leave to later work considerations of fatigue or satiation, both of which can couple the microscopic utilities for working and engaging in leisure. Note, however, that this dependence is for the macroscopic utility function in Eq.(1); other macroscopic utility functions exist in labor supply that do not necessitate this interaction. In general, labor supply theory is concerned with the dependence in the marginal rate of substitution when work and leisure are imperfect substitutes, rather than the macroscopic marginal utilities themselves.

The simplest form for 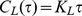 is linear (Fig. 3B, left panel blue line), for which marginal microscopic utility (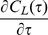) is constant (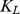, Fig.3B, right panel, blue line). This makes the total microscopic utility of several short leisure bouts the same as that of a single bout of equal total length, and so, just by itself, implies indifference to the division of the duration of a leisure bout. Alternatively, 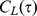 could be concave (e.g., logarithmic, as in Fig. 3B, left panel, red curve). The marginal microscopic utility of leisure would then always decrease as more leisure is consumed (Fig. 3B, right panel, red curve). Subjects should then prefer several short leisure bouts to one long leisure bout. Other non-linear forms are also possible (sigmoidal, quasi concave, see [10]).

A subject's (possibly stochastic) policy (choice-rule) 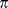 is evaluated according to the average reward rate (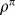), which can be shown to be the ratio of the expected total microscopic utility accumulated during a cycle to the expected total time a cycle takes,
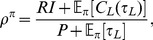
(2)



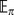
 denotes the expected value under the distribution of leisure durations 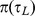 in the post reward-state. The expectation with respect to the policy 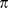 is over a smooth distribution when the policy is stochastic, or is just a point when the policy is deterministic (i.e., the policy is a delta function at a particular leisure duration). The reward-rate increases mostly linearly with reward intensity and decreases mostly hyperbolically with price.

The terminology in reinforcement learning (RL) [18], [21], [25] and optimal foraging [26], [27] concerning the average reward rate differs from that in economics. In RL, 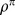 is considered as the opportunity cost per unit time under policy 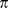. It provides a point of comparison in terms of how lucrative the policy is on average. Committing to performing an action for duration 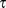 implies forgoing a mean total reward of 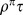. This would be weighed against the benefits of the action. By contrast, in economics, the opportunity cost is defined instead in terms of just the next best action, a quantity that is not very meaningful in our microscopic context. To avoid confusion, we refer to 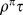 as the average foregone reward (AFR) over period 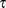.

The (differential) 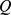-value (see Eq. (A-4) in Text S1) is defined as the expected return of taking action 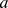 for time 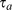 from state 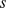, including the immediate microscopic utility, the AFR and the differential value of the next state to which the subject transitions. For engaging in leisure for duration 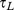 in the post-reward state (using simplified notation), this is
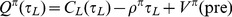
(3)where 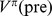 is the differential value of the pre-reward state. Eq. (3) makes clear the distinction between the immediate, innate microscopic utility of leisure 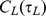 and the net excess return from leisure 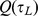. The 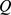-value of working in the pre-reward state can be similarly computed (see Eq. (A-5) in Text S1).

Finally, the 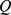-values are used to determine a policy, i.e., a rule for choosing leisure duration 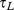. Instead of adopting a descriptive explanation for stochasticity in choice, as for instance in random utility theory, we consider the normative equivalent that starts from the proposition that subjects have a taste for non-deterministic policies 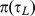. Such a taste is most naturally quantified in terms of the entropy 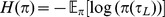. At present, this is merely an assumption; its underpinnings demand careful experimental study. Adopting it makes the problem one of finding
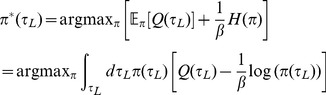
(4)where 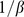 is a temperature parameter that trades off value for entropy. The optimum can be found by computing functional derivatives with respect to 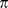 and solving
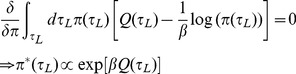
(5)


Appropriately normalizing Eq. (5), we implement 
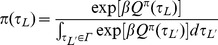
(6)where 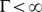 is the range of possible leisure durations. Durations with greater 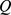-values will be more likely to be chosen. The parameter 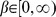 controls the degree of stochasticity in choices: 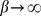 signifies deterministic, optimal choices, while 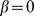 leads to complete uniformity (over the range 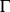 of possible leisure durations). Eq.(6) is called a *softmax* policy; the derivation from a taste for entropy is well-known [28].

#### Model policies

As discussed in [10], we can distinguish various policy regimes. If the payoff is high, then so is the reward rate; thus the AFR 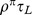 tends to dominate the benefit of leisure 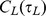 in Eq.(3), no matter what form the latter takes (Fig. 3C, right panels). The probability of duration 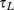 implied by the soft-max policy (Eq.(6)) is then the exponential of a nearly linear function with a steep slope – therefore, an exponential distribution with a short mean (see Sec. A-3 in Text S1). Thus, the subject would work almost continuously, with very short, yet stochastic, exponentially distributed leisure bouts in between work bouts.

At the other extreme, when the payoff is low, the reward rate is small. Consequently, the AFR has a very shallow slope (Fig. 3C, left panels). The 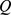-value of leisure then becomes dominated by the microscopic utility of leisure 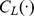. For a linear 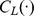, the 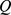-value is still linear, but with a very shallow slope, and the resulting exponential distribution has a long mean (Fig. 3C, left panel, blue curves). For an eventually sub-linear 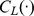, i.e. the marginal utility of which is eventually decreasing, the 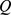-value becomes a unimodal bump. The exponential of this bump yields a unimodal gamma(-like) distribution. If 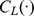 is concave and its marginal microscopic utility does not decrease slowly, the exponential of this bump yields a unimodal gamma(-like) leisure duration distribution with a long tail (Fig. 3C, left panels, red curves). The leisure durations are actually gamma distributed for logarithmic 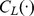 (see Sec A-4 in Text S1).

For intermediate payoffs, the AFR has a slope that is neither too steep nor too shallow (Fig. 3C, middle panels). The 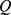-value of leisure depends delicately on the balance between the microscopic utility of leisure and this intermediate AFR.

### Partial allocation with independent marginal utilities

#### Macroscopic utility derived from microscopic utility

To compare our account with that of labor supply theory, we construct a *macroscopic utility* function that is consistent with the microscopic choices *on average*. Consider the case that the subject works for a cumulative amount of time 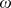, thus completing 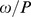 reward and leisure cycles (we allow these to be fractional for simplicity), and is at leisure for a cumulative amount of time 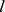. We seek to derive a macroscopic utility function 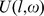 from a microscopic utility function 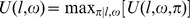, such that the ultimately microscopic choices of durations, and the ultimately macroscopic time allocations are all consistent with the micro-SMDP that we have derived. Here, the notation 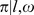 indicates that microscopic choices of leisure duration per cycle have to be consistent with the macroscopic time devoted to leisure on average, i.e., that
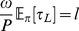
(7)


Consider the microscopic utility
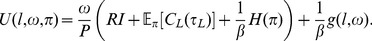
(8)which includes the utilities of the 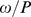 rewards, the expected microscopic utilities of leisure and the entropy, and a function 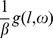, which we will choose to enforce the average foregone reward. We assume 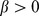 so that the derived utilities are finite. Enforcing Eq. (7) via a Lagrange multiplier 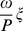, we get
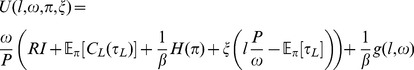
(9)


If we optimise this utility with respect to the policy 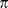, we get
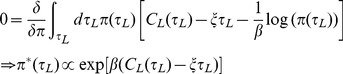
(10)where the Lagrange multiplier 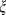 is chosen to satisfy Eq. (7). At this optimum, 
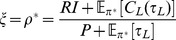
. That is, the Lagrange multiplier or, in economic terms, the “shadow price” (marginal utility of relaxing the constraint in Eq. (7)) is the average reward rate 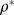. The constructed utility function in Eq. (9) is evaluated at this optimum, and can now be written in terms of macroscopic quantities 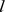 and 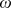 only as
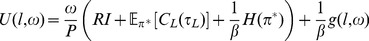
(11)


#### Stochastic microscopic choices

In principle, averaging over stochastic microscopic choices can lead to partial macroscopic time allocation, since the latter concerns the *average* times spent. We now derive this graphically and mathematically, from normative principles. Linear 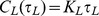 is equivalent to the perfect substitutability case of Eq. (1) with 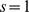, for which deterministic choices exclude partial allocation. However, the derived macroscopic utility in Eq. (11) becomes
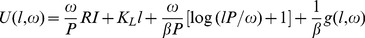
(12)


**Figure 4:**
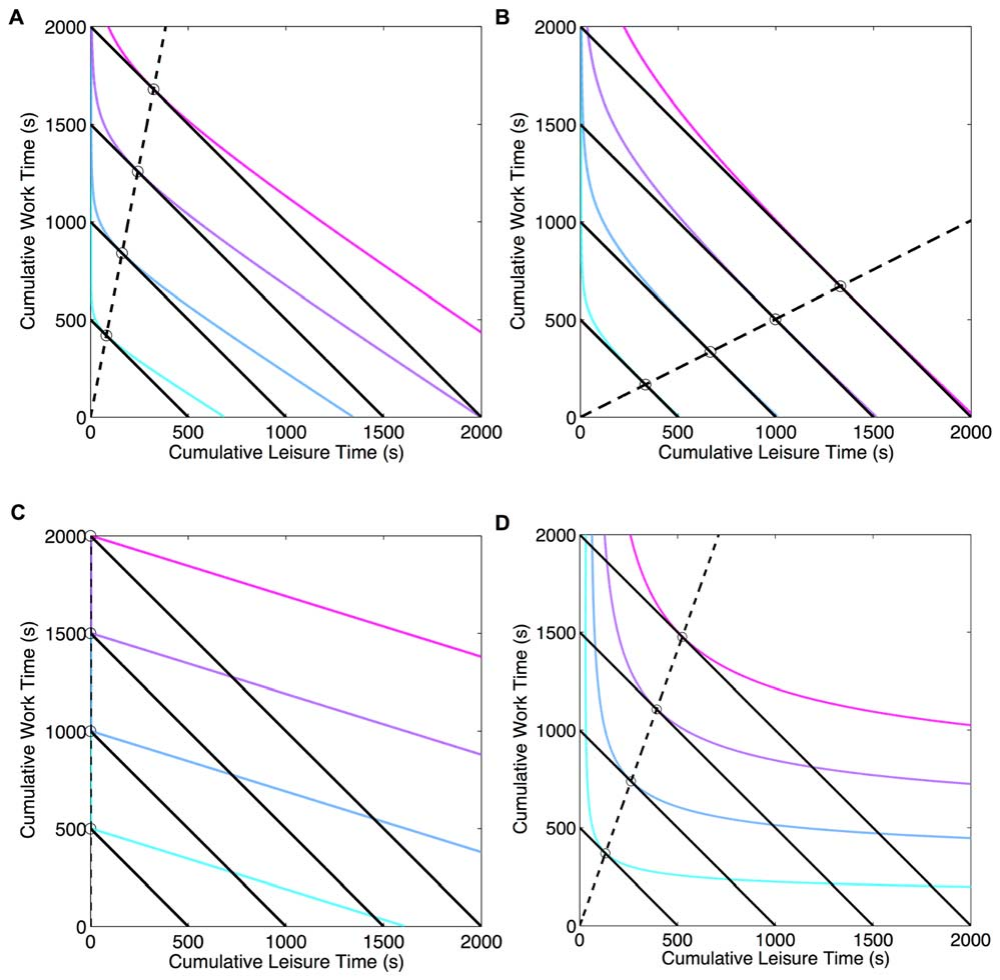
Microscopic choices yield macroscopic partial allocation even with independent marginal utilities. To compare directly with labor supply theory, we *derive* macroscopic utility functions consistent with our assumed microscopic utiities. Curves show indifference curves of the derived macroscopic utility function. Cool colours show order of increasing macroscopic utility. Solid black lines show different budget constraints 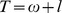 as 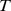 is changed. Dashed black line denotes the path through theoretically predicted optimal leisure and work combinations as 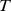 is increased. A), B) Stochastic, approximately optimal microscopic choices with linear 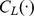 yields partial allocation (A) high and B) medium payoffs are shown). Inverse temperature 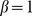. C) Deterministic, optimal microscopic choices with linear 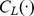 yield all-or-none allocation–work all the time if 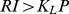. Inverse temperature 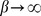. 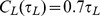, Reward intensity, 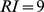 in A), 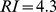 in B) and C), price 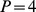
s in A-C. D) Deterministic, optimal choices with non-linear 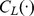 also yields partial allocation. 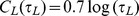, 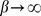, 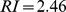 and price 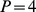
s.

Its ICs have negative slopes, which, for stochastic choices (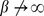), are not constant. These changes in slope generate partial time allocations (Fig.4A,B), when a budget constraint (BC; solid black lines) is tangent to an IC. Including an appropriate 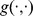 (Eq. (A-14) in Text S1) enables the optimal macroscopic combination of cumulative work and leisure times to be consistent with the microscopic mean leisure duration. At the optimum, 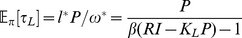 as long as 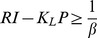, and 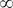 otherwise (Eqs. (A-9), (A-10) in Text S1). Thus stochasticity replaces substitutability in generating partial allocation.

For 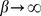, optimal microscopic choices are purely deterministic. The derived utility function in Eq.(12) becomes
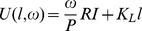
(13)which directly corresponds to the utility function of labor supply theory in Eq.(1) with 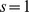 and would lead to total allocation to work or leisure depending on whether work or leisure is more beneficial, i.e. the sign of 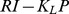 (Fig. 4C; compare with Fig. 1, upper- panels).

#### Deterministic, optimal microscopic choices

As for standard labor supply theory, the assumption of stochasticity is not necessary to achieve partial allocation if the microscopic utility of leisure is a suitably non-linear function of its duration, e.g., the concave 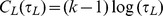, for 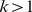 (Fig. 3B, red). Choosing concave 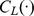 is for convenience; it would further be straightforward to take 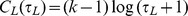 so that the microscopic utility is defined over all 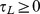. Importantly, though, the *microscopic marginal utility* of leisure need not depend on the amount of work done. For a deterministic policy (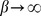), the derived macroscopic utility function (see Eq. (A-16) in Text S1)is
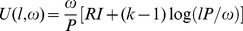
(14)for which the slopes of the (*macroscopic*) ICs depend on the amount of work and leisure accumulated (Fig. 4D) and generate partial allocation as optimal solutions. Thus, neither stochasticity nor an interaction between work and the marginal utility of leisure is necessary for partial allocation.

### Traditional macroscopic accounts: II

#### Generalized matching law: Mountain model

An alternate macroscopic characterisation of behavior that yields smooth time allocation curves, hypothesises that subjects match (according to the generalised matching law, [4], [29]) their time allocation between work and leisure to the ratio of their payoffs [29], 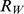 and 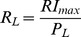, respectively [2], [30]

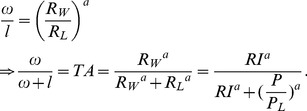
(15)


**Figure 5:**
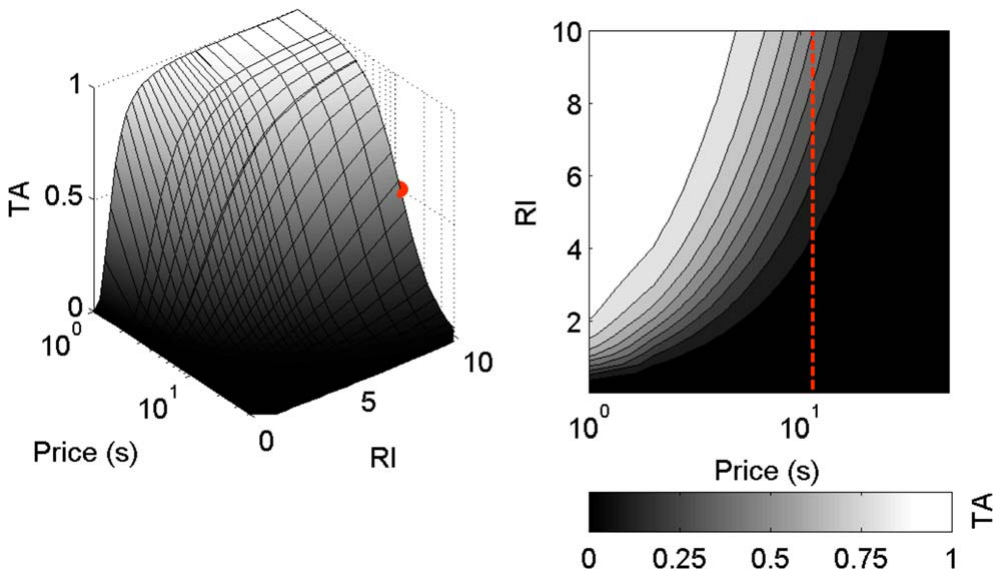
Mountain model. Left panel: 3-dimensional relationship; right panel: contours of equal time allocation, as a function of reward intensity and price predicted by the mountain model using the generalised matching law. Red lines in right panel show 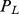: the price at which 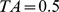 for a maximal reward intensity (red dot in left panel). 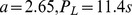. The *TA* contours smoothly increase with reward intensity and smoothly decrease with price.

Here, 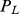 is defined as the price at which, for a maximum subjective reward intensity 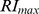, the subject allocates half the time to work, and half to leisure (see red lines in Figs.5 and S2A).

This establishes a 3-dimensional relationship between *TA*, *subjective* reward intensity and price (Fig.5, left panel) that is analogous to the *mountain model* [12], [31]), which plots this relationship in terms of the *objective* reward strength. *TA* is smooth, and increases and decreases monotonically with reward intensity and price, respectively, as evident in the contours in Fig. 5 (right panel). Stochastic *macroscopic* allocation, by virtue of generalised matching, therefore accounts for partial time allocation. The matching coefficient 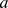 determines how *TA* increases as a function of the payoff from work – rapidly for over-matching (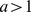), and slowly for under-matching ((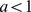), Fig. S2B, respectively).

### The microscopic mountain

**Figure 6:**
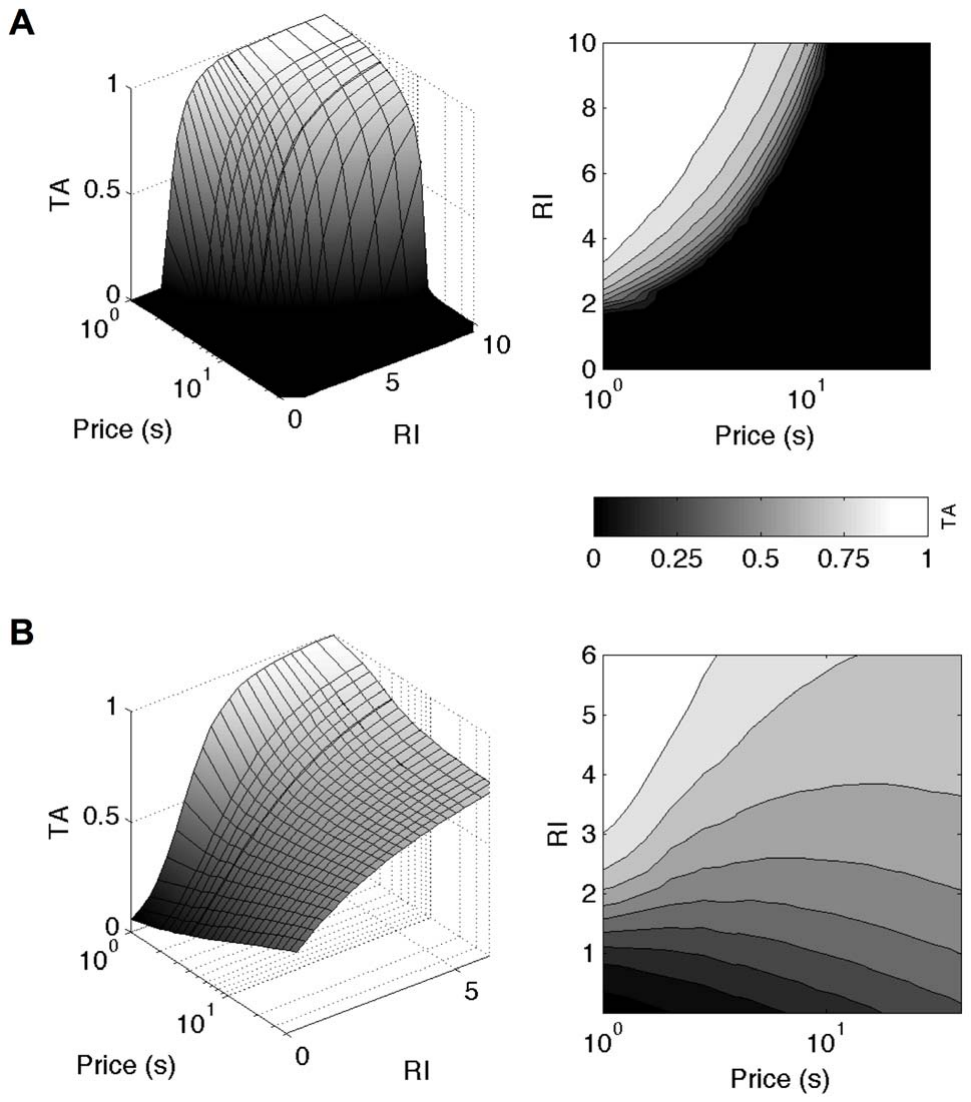
Macroscopic time allocation derived from normative, microscopic choices yields a superset of the mountain model. Left panels: 3-dimensional relationships between *TA*, reward intensity and price, right panel: contours of equal *TA*, predicted by the micro SMDP model for A) linear, B) concave 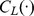. The 3-dimensional relationship and smooth contours for a linear 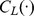 derive the mountain model in Fig.3. Note that an extra, higher set of reward intensities was necessary to achieve the full range of time allocation for linear 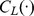. The fact that contours change direction at longer prices for a non-linear 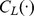 rather than decrease monotonically reflects that *TA* may no longer decrease and even increase as the price is increased further.

By integrating the microscopic choices from our model, we can compare it with macroscopic descriptions such as the mountain model. We saw that linear 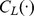 generates partial allocation with stochasticity. It therefore generates smooth (non-step function) macroscopic time allocation curves as a function of both reward intensity and price. Consequently, 3-dimensional relationships can be derived that are qualitatively similar to those specified by the mountain model (when expressed in terms of subjective reward intensity, compare Fig. 6A with Fig. 5).

**Figure 7:**
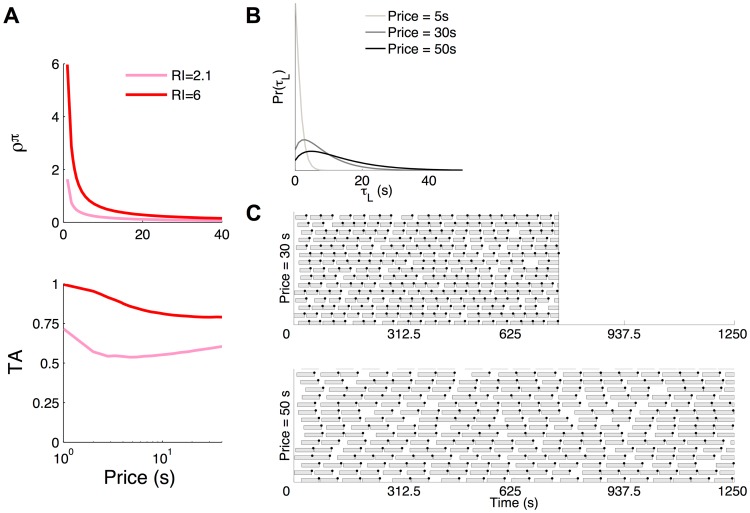
Time allocation may not decrease with price for a non-linear microscopic utility of leisure. A) Upper panel: Reward rate (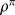) and lower panel: time allocation (*TA*) for a concave microscopic utility of leisure as a function of price. A small and a high reward intensity are shown. Reward rate decreases hyperbolically with price, eventually asymptoting. B) Leisure duration distribution as a function of price for a fixed high reward intensity (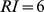). At very long prices, as the price is increased further (eg. from 30 s to 50 s), the mode of the leisure duration distribution does not change by much although the mean does. C) Ethograms for two long prices. As price is increased, the work bouts (proportional to the price) do increase. Leisure bouts, drawn from the mode, do not change by much. Consequently, *TA* no longer decreases but may even increase with price (A, lower panel). This is despite the trial duration being normalised to a multiple (here 25) of the price. It is the lack of significant change in the majority of leisure durations that is critical. We normalised by the trial duration of 25 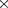 price, instead of simply normalizing by the price, to emphasise that *TA* is a macroscopic quantitity and to be consistent with the procedure in the example data Figure S1.

However, when 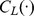 is non-linear, more complicated structures arise. If the price is increased while holding the reward intensity fixed, the reward rate 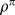 (Eq. (2)) decreases hyperbolically and eventually asymptotes (Fig.7A). Consequently, unlike the mean, the mode of the gamma-like distribution does not substantially increase with the price (see Figs.3C and 7B). Since the mode determines the duration of the majority of leisure bouts, these do not increase substantially. If the subject continues to work for the entire price duration (Fig.7C), then, surprisingly from the macroscopic perspective of the generalized matching model, the total work time and thus the *TA* will *increase*, rather than decrease with the price (Figs.6B and 7A, lower panel). This prediction is readily amenable to experimental test.

Since for linear 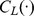, leisure durations are governed by substantially changing means and not modes, *TAs* are in general smaller than for strictly concave 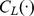, implying that higher payoffs are necessary to capture the entire *TA* range.

## Discussion

We studied the problem of partial time allocation – when reward intensities and prices are not extreme, both animals and humans divide their time between work and leisure. Traditional theories such as the microeconomic theory of labor supply, or accounts from behavioral psychology based on the generalised matching law, have characterised behavior at a macroscopic level, studying average times spent in work or leisure. While labor supply approaches have studied choices within periods of time, these have been limited to maximising utility within these time windows [32]–and thus, still average times within these windows. We proposed a normative, microscopic approach using the reinforcement learning framework of Semi-Markov Decision Processes. Although we applied it to the labor-leisure tradeoff, this is actually a more general theoretical framework for temporally relevant decision-making. By integrating the microscopic choices of our model over time, we were able to account for the nature of macroscopic partial allocation.

We showed how assumptions about microscopic and macroscopic quantities relate. In labor supply theory, the marginal utility of leisure may (although not necessarily) depend on the amount of work (or rewards) consumed, and (unlike in the behavioral data) choices are classically deterministic. We considered a stochastic policy of the same form as emerges for standard random utility models, but directed at microscopic, rather than macroscopic, choices. Macroscopic random utility theory considers stochasticity to be due to unobservable noise, which is added to the representation of utility. The subject chooses the combination of cumulative work and leisure times that maximizes this net utility (including the noise term). If the noise is assumed to be Gumbel distributed (i.e. drawn from an extreme value distribution of type I), then the probability of choosing the optimal combination is a softmax. The softmax function that we employ is over microscopic durations, and arises from an (equivalently arbitrary) assumption that subjects have a taste for entropic policies. Randomness is thus directly built into the fabric of our model, rather than being an afterthought. It generates partial allocation even when the marginal microscopic utility of leisure is independent of work.

Previous exercises attempting to link macroscopic static and dynamic frameworks have not been generally successful [9]. Optimal choice in a dynamic context generally depends on the microscopic state, whose evolution is invisible at a macroscopic level. This allows the macroscopic average choice obtained after integrating out such states (i.e., the average choice under the stationary distribution) to appear counterintuitive, possibly even violating rationality constraints. In our case, the key feature of the microscopic state is implicit in the non-memorylessness of the policies allowed in an SMDP – e.g., that the hazard function governing the probability a leisure bout will end a certain time after it begun is not independent of time.

An example of the problems comes from observing that time allocation to working under conventional macroscopic labor supply accounts generally increases with reward and decreases with price. Something similar is true of the macroscopic, mountain-like, consequence of generalized matching. We showed in our framework that, although this can be true, it is nevertheless the case that for certain non-linearities, the time allocated to working can increase rather than decrease as the price increases, yielding complicated 3-dimensional relationships and non-monotonic contours that elude the mountain model. We thus derived a transparent link between microscopic and macroscopic frameworks. Whereas animals have been previously shown consistently to work more when work-requirements are greater (one idea is that this arises from sunk costs [33], [34]), the apparent anomaly discussed here only occurs at longer prices and is due to the form of the microscopic utility of leisure. This is an obvious candidate for empirical investigation [35].

Non-linear benefit of leisure functions can also lead to partial allocation for deterministic choices. This applies even for functions that differ from those common in labor supply theory in virtue of satisfying independence between the microscopic utilities of working and engaging in leisure. Of course, the marginal microscopic utility of leisure might depend on work or rewards – for instance due to fatigue or satiation. However, carefully eliminating such dependencies (by, e.g., allowing subjects sufficient rest inbetween trials, and using non-satiating rewards like BSR) may provide an avenue to quantify aspects of the microscopic utility of leisure empirically. This should help reveal why and how subjects partially allocate their time. It would then be natural to extend the study to considerations of effort, fatigue and cognitive computational costs [36]–[40] (e.g. from holding down weighted levers or performing cognitively demanding tasks) and the effects of manipulating motivational state [12], [41], [42]. It is by taking advantage of the greater precision available from the detailed topography of work and leisure that we may hope to gain insight into these most important details. Although previous work has described aspects of this topography [37], [43], our precise control theoretic formalization could offer enrichment.

The utilities considered in macroscopic labor supply theory are ordinal, whereas the microscopic utilities used in our framework are cardinal and, by analogy with quantities investigated in discrete choice paradigms [22]–[24], open for direct neural investigation. One of the key goals of our work is to provide a formal framework within which this can happen.

Finally, our work provides a foundation for studying critical psychological processes and neural computations at an appropriate timescale. Real-time or quasi-real-time recording methods in routine use in neuroscience such as electrophysiology, large-scale imaging, or fast-scan cyclic voltammetry allow us to correlate the activity of neural populations or concentrations of neuromodulators with the execution of behaviors. Likewise, fast causal manipulations via such methods as optogenetics allow the circuits governing these behaviors to be probed in a highly selective manner. There is an evident mismatch between the microscopic timescale over which these methods operate and the macroscopic timescales over which (a) behavior has often been characterised; and (b) the quantities such as costs and benefits which underpin the pertinence of the behavior have been defined. Our normative microscopic account may therefore provide an illuminating framework within which to build explanations that span multiple levels.

## Methods

See Micro-SMDP methods in Text S1.

## Supporting Information

Figure S1
**Partial time allocation: example task and data.** A) Cumulative handling time (CHT) task. Grey bars denote work (e.g. holding down a lever), white gaps show leisure (eg. grooming, resting, sleeping etc.). The subject must accumulate work up to a total period of time called the *price* (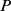) in order to obtain a single reward (black dot) of subjective reward intensity 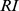. The trial duration is 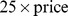. The reward intensity and price are held fixed within a trial. B) Macroscopic time allocation (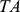) functions of a typical subject as a function of reward intensity and price. Red curves: effect of reward intensity, for a fixed short price; blue curves: effect of price, for a fixed high reward intensity; green curves: joint effect of reward intensity and price. C) Microscopic *ethogram* showing the detailed temporal topography of working and engaging in leisure for the subject in B) for a medium payoff respectively, for a fixed, short price. The part of a trial before the reward and price are certainly known is coloured pink and not considered further. Data initially reported in [13], [44].(TIF)

Figure S2
**Mountain model parameters**. Left 3-dimensional relationship; right panel: contours of equal time allocation, as a function of reward intensity and price predicted by the mountain model using the generalised matching law. Red lines in right panels show 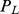: the price at which 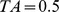 for a maximal reward intensity (red dot in left panels). A) For a small 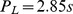, while overmatching 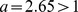 as in the main text and B) undermatching 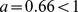 while 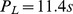 as in the main text.(TIF)

Text S1 **Supporting information.**
(PDF)
