# Supplementary figures and images for: Some Work and Some Play: Microscopic and Macroscopic Approaches to Labor and Leisure
Source: PLoS Comput Biol. 2014 Dec 4;10(12):e1003894. doi: 10.1371/journal.pcbi.1003894 (PMC4256012; doi:10.1371/journal.pcbi.1003894)

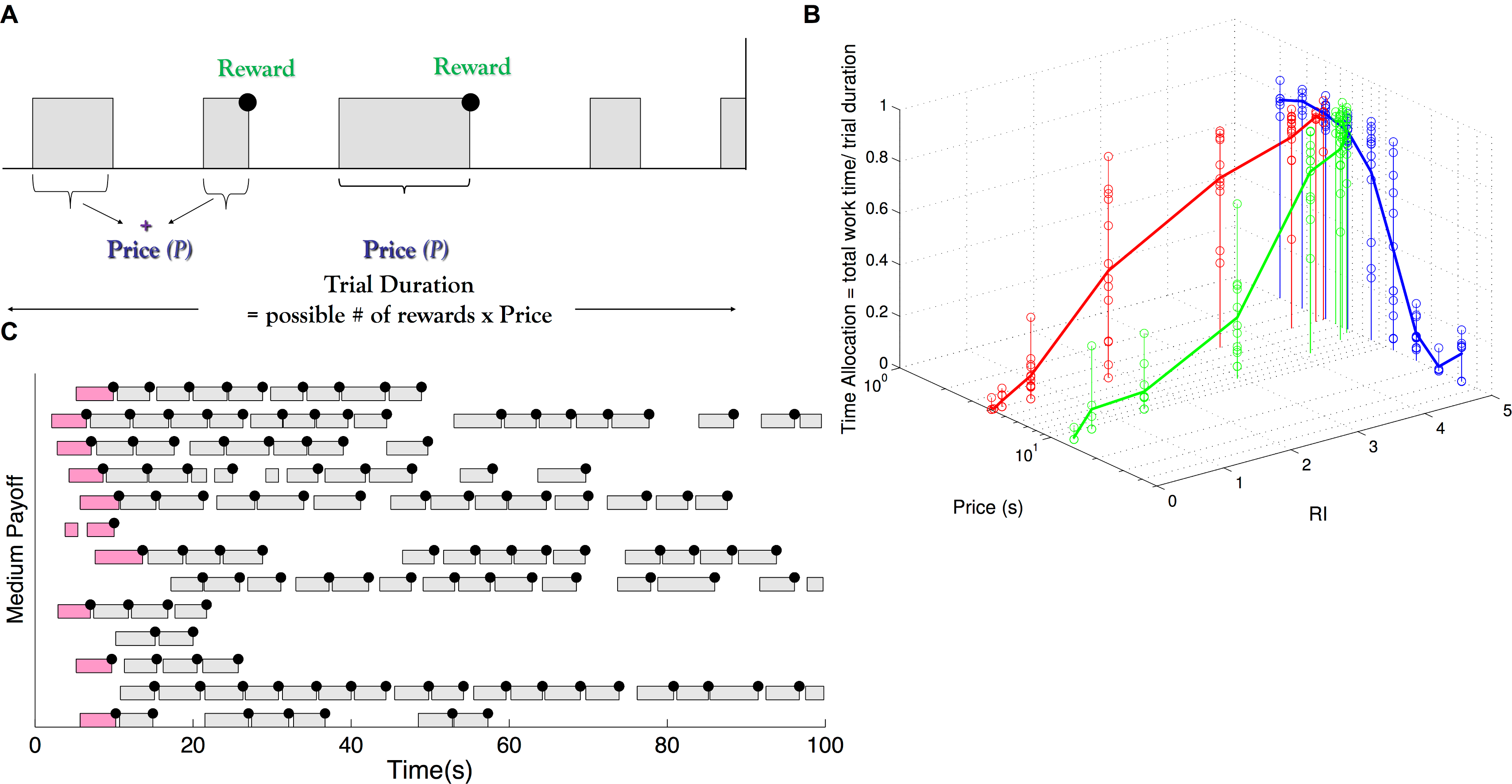

Supplement: Figure S1 — Partial time allocation: example task and data. A) Cumulative handling time (CHT) task. Grey bars denote work (e.g. holding down a lever), white gaps show leisure (eg. grooming, resting, sleeping etc.). The subject must accumulate work up to a total period of time called the price () in order to obtain a single reward (black dot) of subjective reward intensity . The trial duration is . The reward intensity and price are held fixed within a trial. B) Macroscopic time allocation () functions of a typical subject as a function of reward intensity and price. Red curves: effect of reward intensity, for a fixed short price; blue curves: effect of price, for a fixed high reward intensity; green curves: joint effect of reward intensity and price. C) Microscopic ethogram showing the detailed temporal topography of working and engaging in leisure for the subject in B) for a medium payoff respectively, for a fixed, short price. The part of a trial before the reward and price are certainly known is coloured pink and not considered further. Data initially reported in [13], [44]. (TIF) [file pcbi.1003894.s001.tif]

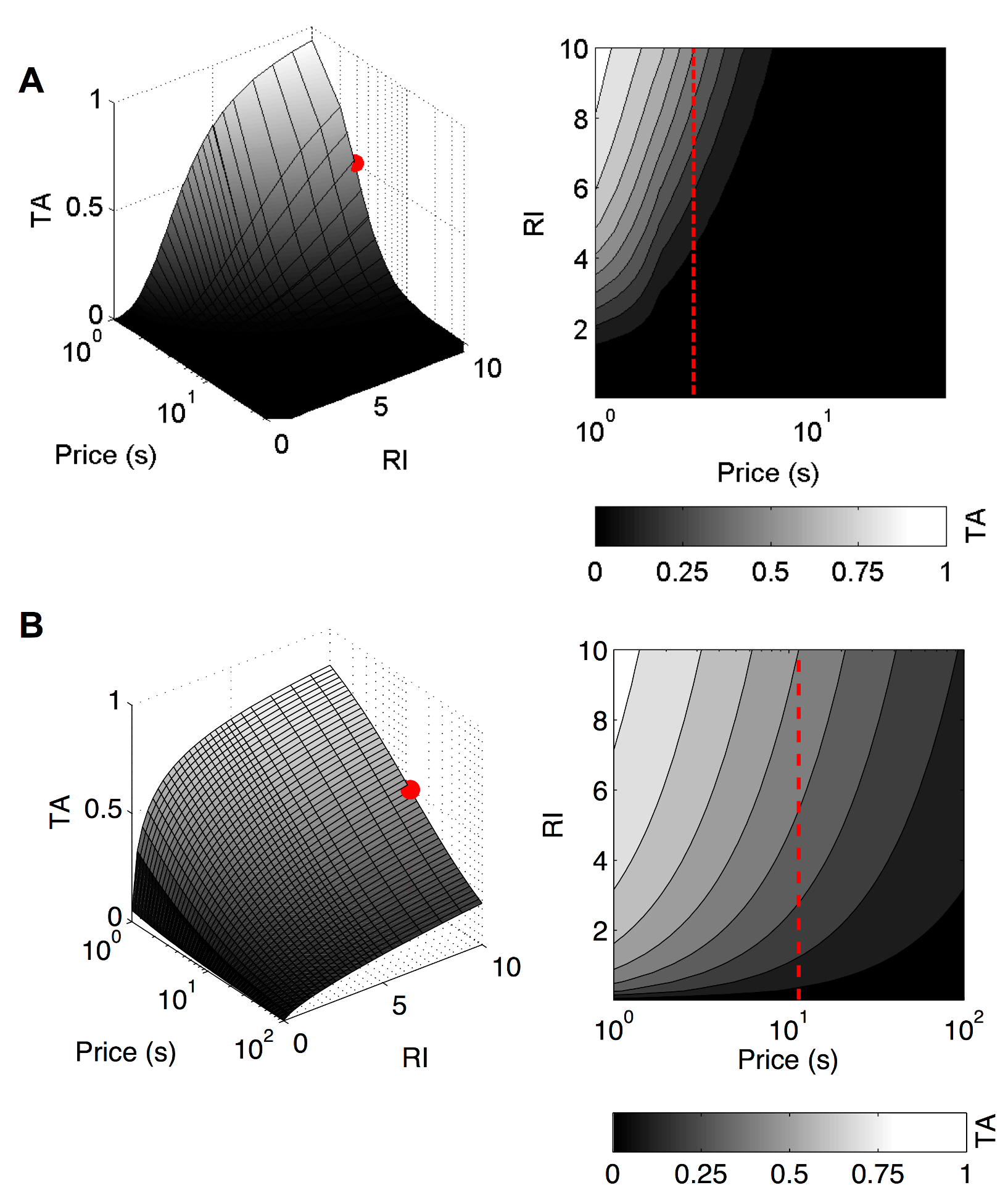

Supplement: Figure S2 — Mountain model parameters. Left 3-dimensional relationship; right panel: contours of equal time allocation, as a function of reward intensity and price predicted by the mountain model using the generalised matching law. Red lines in right panels show : the price at which for a maximal reward intensity (red dot in left panels). A) For a small , while overmatching as in the main text and B) undermatching while as in the main text. (TIF) [file pcbi.1003894.s002.tif]
