# Supplementary material for: Some Work and Some Play: Microscopic and Macroscopic Approaches to Labor and Leisure
Source: PLoS Comput Biol. 2014 Dec 4;10(12):e1003894. doi: 10.1371/journal.pcbi.1003894 (PMC4256012; doi:10.1371/journal.pcbi.1003894)
Supplement: Text S1 — Supporting information. (PDF) [file pcbi.1003894.s003.pdf]

Text S1
Some work and some play: microscopic and macroscopic approaches to labor and leisure

Ritwik K. Niyogi[1,*], Peter Shizgal[2], Peter Dayan[1]

1 Gatsby Computational Neuroscience Unit, University College London, London, United Kingdom

2 Center for Studies in Behavioral Neurobiology, Concordia University, Montreal, Quebec, Canada

∗ **E-mail: ritwik.niyogi@gatsby.ucl.ac.uk**

A-1 Macroscopic time allocation from labor supply theory

If we consider the rewards to be continuous (instead of quantised: delivered exactly when the price is attained) or if we consider expected times spent in work or leisure only, we can construct a budget constraint (BC): the total amount of work ω and leisure l is the trial duration T

$$\omega + l = N\,P + l = T \tag{A-1}$$

where P and N are the price and number of rewards earned, respectively. Note that this budget constraint is linear in N and l.

In general, given that we maximise macroscopic utility (according to the function in Eq.(1)) subject to a BC, we can derive the time allocation

$$TA = \frac{(\frac{RI}{K_L})^{-\frac{1}{s-1}}}{(\frac{RI}{K_L})^{-\frac{1}{s-1}} + \; P^{\frac{s}{1-s}}} \tag{A-2}$$

which increases with RI and (for $s \geq 0$) decreases with price (Figure 2)

A-2 Micro SMDP methods

We formulate our model as a infinite-horizon (unichain) Semi-Markov Decision Process (SMDP) [1]. A state s contains all the information necessary for making a decision. The subject's next state in the future S' depends on its current state S, the action a, and the duration τ_a of that action, but is independent of all other states, actions and durations in the past. We further assume subjects jointly choose both the actions and their durations, as in [2–4]. We discretize leisure durations to 1s time-bins in our software implementations, but make sure that our results are the same for even finer discretizations. Note that when choosing an action a for duration τ_a, the subject commits to executing this action to its completion, rather than choosing actions at each moment in time. An alternative to our model is one in which choices are made at the finest possible temporal granularity rather than having determinable durations. So a duration of leisure is equivalent to a sequence of 'leisure-leisure-leisure' choices. We have called this a 'nanoscopic' model [5], and noted its straightforward formal relationship to our microscopic SMDP

model. The distinction between these formulations cannot be made behaviorally, but may be possible in terms of their neural implementations.

A choice rule or *policy* $\pi([a,\tau_a]|S)$ specifies the subject's probability of taking action a for time τ_a in state S. Under a given policy, we can define the expected reward rate, or the average reward per unit time

$$\rho^\pi = \lim_{\mathrm{T}\to\infty} \frac{\mathbb{E}_\pi\left[\sum_{t'=0}^{\mathrm{T}-1} r^+_{t'}([a_{t'},\tau_{a_{t'}}]) - r^-_{t'}([a_{t'},\tau_{a_{t'}}])\right]}{\mathrm{T}} \tag{A-3}$$

where $r^+_{t'}$ and $r^-_{t'}$ denote the benefits and costs at time points t'. Note that the expected reward rate is independent of the starting state. Suppose $\hat{r}^+(S,[a,\tau_a])$ and $\hat{r}^-(S,[a,\tau_a])$ are the expected benefits and costs of taking action a, for duration τ_a from state S.

Normatively, a subject should try to (approximately) maximise its expected return. The expected return or (differential) Q-value of taking action a, for duration τ_a from state S is

$$\begin{aligned} Q^\pi(S,[a,\tau_a]) &= \mathbb{E}_\pi\left[\sum_{t'=0}^{\infty} (r^+_{t'}([a_{t'},\tau_{a_{t'}}]) - r^-_{t'}([a_{t'},\tau_{a_{t'}}]) - \rho^\pi\tau_{a_{t'}})\,|S_t = S, a_t = a, \tau_{a_t} = \tau_a\right] \\ &= \hat{r}^+(S,[a,\tau_a]) - \hat{r}^-(S,[a,\tau_a]) - \rho^\pi\tau_a + V^\pi(S') \\ &= \hat{r}^+(S,[a,\tau_a]) - \hat{r}^-(S,[a,\tau_a]) - \rho^\pi\tau_a + \sum_{a'}\int_{\tau_{a'}} \pi([a',\tau_{a'}]|S')\, Q^\pi(S',[a',\tau_{a'}])\, d\tau_{a'} \end{aligned} \tag{A-4}$$

where $V^\pi(S) = \sum_a \int_{\tau_a} \pi([a,\tau_a]|S)\, Q^\pi(S,[a,\tau_a])$ is the *value* of state S, averaged across all actions and their times. The subject forgoes average reward $\rho^\pi\tau_a$[1] for taking action a for time τ_a [2–4].

Other choices of optimization criterion would be possible – notably an exponentially discounted return. However, it is conventional to use the long-run average reward in recurrent problems such as this [2, 6], particularly given that macroscopic measurements are themselves typically also couched in terms of rates (in this case, of responding). Further not only does using the average reward obviate the requirement to set the discount rate, but also it is known that sufficiently shallow exponential discounting leads to exactly the same policy as the average reward [1, 7].

While simultaneously solving Eqs. (S-3) and (S-4) for the reward rate and the Q-values, we have more unknowns than equations. As conventional, we therefore set the value of one of the states to 0, and solve for the Q values relative to this baseline. The Q values reported here are therefore *differential* and not the actual ones. We drop differential denotations and simply refer to them as Q-values. Since the policies depend on Q-values, which themselves recursively depend on the policies, except in the case of the optimal policy, we cannot solve for them in closed form. We use policy iteration to find them [1, 8].

For simplicity, we assume that if the subject works, then it works continuously for the entire duration of the price, after which it engages in leisure in the post-reward state. The Q-value of working in the pre-reward state then comprises: (i) the reward of reward intensity RI, (ii) an AFR $\rho^\pi P$, and (iii) the value of the post-reward state

[1] This is characterized in reinforcement learning terms as an automatic *opportunity cost of time*.

$$Q^{\pi}(\text{pre}, [W, P]) = RI - \rho^{\pi} P + V^{\pi}(\text{post}). \tag{A-5}$$

Since we define leisure to be possible in the post-state only, we simplify notation by dropping the "post" and $[L, \tau_L]$ denotations in $\pi([L, \tau_L]|\text{post})$ and $Q(\text{post}, [L, \tau_L])$, and simply use $\pi(\tau_L)$ and $Q(\tau_L)$.

A-3 Linear microscopic utility of leisure yields exponentially distributed leisure durations

Suppose the microscopic utility of leisure is linear, $C_L(\tau_L) = K_L \tau_L$. Then the Q-value of engaging in leisure in the post-reward state is also linear, $Q^{\pi}(\tau_L) = (K_L - \rho^{\pi})\tau_L + V^{\pi}(\text{pre})$. According to the softmax policy, the probability of choosing to engage in leisure for time τ_L in the post-reward state is proportional to the exponential of the Q-value. This probability is $\pi(\tau_L) \propto \exp\left[-\beta(\rho^{\pi} - K_L)\tau_L\right]$, which is an exponential distribution with mean $\mathbb{E}[\tau_L] = \frac{1}{\beta(\rho^{\pi} - K_L)}$. Thus, for linear $C_L(\cdot)$, leisure bout durations are always exponentially distributed with a mean which depends on the reward rate. The greater the reward rate, the shorter is the mean leisure bout.

A-4 Logarithmic microscopic utility of leisure yields gamma distributed leisure durations

For a logarithmic microscopic utility a leisure $C_L(\tau_L) = (k-1)\log(\tau_L)$; the Q-value of engaging in leisure in the post-reward state is a unimodal bump. The leisure duration distribution is the exponential of this bump: $\pi(\tau_L) = \frac{1}{\Gamma(1/\beta\rho^{\pi})} \tau_L^{\beta(k-1)} \exp(-\beta\rho^{\pi}\tau_L)$. This is a gamma distribution with shape parameter $\bar{k} = \beta(k-1) + 1$ and scale parameter $\frac{1}{\beta\rho^{\pi}}$. The mode of this gamma distribution is $\frac{k-1}{\rho^{\pi}}$. Thus, if the reward rate does not change substantially, neither does this mode. For the special case, of $k = 1$, the gamma distribution becomes an exponential distribution.

A-5 Reward rate and mean leisure duration for a linear microscopic utility of leisure

For a linear $C_L(\cdot)$, the reward rate and mean leisure duration can be analytically, self-consistently derived. The reward rate in Eq.(2) is simply,

$$\rho^{\pi} = \frac{RI + K_L \mathbb{E}[\tau_L]}{P + \mathbb{E}[\tau_L]} \tag{A-6}$$

As discussed above, leisure durations in the post-reward state are exponentially distributed with mean

$$\mathbb{E}[\tau_L] = \frac{1}{\beta(\rho^{\pi} - K_L)} \tag{A-7}$$

Re-arranging terms of this equation,

$$\rho^{\pi} = \frac{1}{\beta\,\mathbb{E}[\tau_L]} + K_L \tag{A-8}$$

Equating Eqs. (S-6) and (S-8) and solving for the mean leisure duration $\mathbb{E}[\tau_L|\text{post}]$, we derive

$$\mathbb{E}[\tau_L] = \frac{P}{\beta(RI - K_L P) - 1} \tag{A-9}$$

This is the mean leisure duration as long as $RI - K_L P > 1/\beta$, and $\mathbb{E}[\tau_L] \to \infty$ otherwise. When the former condition holds, we may substitute Eq. (S-9) into Eq. (S-6) and solve for ρ^{π}

$$\begin{aligned} \rho^{\pi} &= \frac{(RI - K_L P)\ (\beta RI - 1)}{(RI - K_L P)\ \beta P} \\ &= \frac{\beta RI - 1}{\beta P} \end{aligned} \tag{A-10}$$

A-6 Macroscopic utility derived from linear and non-linear microscopic utilities

The point of the utility function in Eq. (11) is to lead to choices whose macroscopic characterization is the same as those of the micro-SMDP. In particular, this means that if we maximize $U(l, \omega)$ subject to a budget constraint $l + \omega = T$ for some total duration T, then we will recover what we know to be true of the optimum $l^* P/\omega^* = \mathbb{E}_{\pi}[\tau_L|\text{post}] = \frac{P}{\beta(RI - K_L P) - 1}$ (Eq.(S-9)). Given the form of the optimal microscopic policy associated with Eqs.(3) and (4), we also require that the Lagrange multiplier ξ associated with Eq.(10) should take on the value $\rho^* = \frac{RI + \mathbb{E}_{\pi^*}[C_L(\tau_L)]}{P + \mathbb{E}_{\pi^*}[\tau_L]}$.

As required for macroscopic utility functions considered in economics, macroscopic utilities with respect to both work (or rewards) ($\frac{\partial U}{\partial \omega}$) and leisure ($\frac{\partial U}{\partial l}$) are positive. Then, since macroscopic utility is constant on an indifference curve, the total derivative with respect to a good (say leisure) is zero:

$$\begin{aligned} \frac{dU}{dl} &= \frac{\partial U}{\partial l} + \frac{\partial U}{\partial \omega}\frac{d\omega}{dl} = 0 \\ \Rightarrow \frac{d\omega}{dl} &= -\frac{\partial U}{\partial l}\Big/\frac{\partial U}{\partial \omega} < 0 \end{aligned} \tag{A-11}$$

This shows that indifference curves have negative slopes ($\frac{d\omega}{dl} < 0$).

The optimum (l^*, ω^*) associated with the budget constraint occurs when

$$\begin{aligned} \frac{d\omega}{dl}\Big|_{(l^*,\omega^*)} &= \frac{d(T - (\omega + l))}{dl}\Big|_{(l^*,\omega^*)} = -1 \\ \Rightarrow \frac{\partial U}{\partial l}\Big|_{(l^*,\omega^*)} &= \frac{\partial U}{\partial \omega}\Big|_{(l^*,\omega^*)} \end{aligned} \tag{A-12}$$

Consider the case of a linear microscopic utility of leisure, $C_L(\tau_L) = K_L \tau_L$. In this case, the optimum π^* of Eq.(10) is exponential, implying that $\pi^*(\tau_L)|l, \omega = \frac{\omega}{lP}\exp\left[-\frac{\omega}{lP}\,\tau_L\right]$ whose entropy $H(\pi^*) =$

$\log(lP/\omega) + 1$. Consequently, the derived macroscopic utility function in Eq.(11) becomes

$$U(l,\omega) = \frac{\omega}{P} RI + \; K_L \, l + \frac{\omega}{\beta \, P} \left[\log(lP/\omega) + 1 \right] + \frac{1}{\beta} \, g(l,\omega) \tag{A-13}$$

If we define

$$g(l,\omega) = \frac{1}{P} \left[\, l(\log(l) - 1) + \omega(\log(\omega) - 1) - \omega(\log(P) + 1) \, \right] = \frac{\omega + l}{P} [\log(l) - 1] - \frac{\omega}{P} H(\pi^*) \tag{A-14}$$

Then it turns out that the optimum has just the correct properties in terms of choice. We merely claim that this a possible $g(\cdot,\cdot)$ – it need not be unique.

If, instead, the microscopic utility of leisure is logarithmic: $C_L(\tau_L) = (k-1)\log(\tau_L)$, then, as in the case for linear $C_L(\cdot)$, we can derive $\mathbb{E}_\pi[C_L(\tau_L)]$ and $H(\pi)$ analytically in closed form for policies associated with Eq.(10).

$$\begin{aligned} \mathbb{E}_\pi[C_L(\tau_L)] &= (k-1)\mathbb{E}_\pi[\log(\tau_L)] = (k-1)[\psi(\bar{k}) + \log(\mathbb{E}_\pi[\tau_L]) - \log(\bar{k})] \\ H(\pi) &= \log(\mathbb{E}_\pi[\tau_L]) - \log(\bar{k}) + \bar{k} + (1-\bar{k})\psi(\bar{k}) + \log(\Gamma(\bar{k})) \end{aligned} \tag{A-15}$$

Here $\Gamma(\cdot)$ and $\psi(\cdot)$ represent the *gamma* and *digamma* functions, respectively and $\bar{k} = \beta(k-1)+1$ as above. It is easy to see that for the special case of $k=1$, i.e. when the gamma distribution becomes an exponential distribution, $\bar{k}$ becomes simply 1. In that case, $H(\pi) = \log(\mathbb{E}_\pi[\tau_L]) + 1$ as above, since all other quantities in Eq.(S-15) vanish. Further, if we considered a general microscopic utility which was a sum of logarithmic and linear components, $\hat{C_L}(\tau_L) = (k-1)\log(\tau_L) + K_L\tau_L$, then we could treat the linear version as a special case by simply setting $k=1$. Using the quantities in Eq.(S-15), we may derive a macroscopic utility from a microscopic logarithmic utility

$$\begin{aligned} U(l,\omega) &= \frac{\omega}{P} \left(RI + \; (k-1) \, \mathbb{E}_{\pi^*}[\log(\tau_L)] + \frac{1}{\beta} \, H(\pi^*) \right) + \frac{1}{\beta} \, g(l,\omega) \\ &= \frac{\omega}{P} \left(RI + \; (k-1) \left[\psi(\bar{k}) + \log\left(\mathbb{E}_{\pi^*}[\tau_L]\right) - \log(\bar{k}) \right] \right) + \frac{1}{\beta} \left[\frac{\omega}{P} H(\pi^*) + \; g(l,\omega) \right] \\ &= \frac{\omega}{P} \left(RI + \; (k-1) \left[\psi(\bar{k}) + \log\left(l \, P/\omega\right) - \log(\bar{k}) \right] \right) + \frac{1}{\beta} \left[\frac{\omega}{P} H(\pi^*) + \; g(l,\omega) \right] \end{aligned} \tag{A-16}$$

then, if

$$g(l,\omega) = \frac{1}{P} \left[\, l(\log(l) - 1) + \omega(\log(l) - 1)) \, \right] - \frac{\omega}{P} H(\tau_L) = \frac{\omega + l}{P} [\log(l) - 1] - \frac{\omega}{P} H(\pi^*) \tag{A-17}$$

then one can show that not only does maximising the derived macroscopic utility in Eq.(S-16) subject to a BC yield the appropriate mean leisure duration: $\mathbb{E}_\pi[\tau_L|\text{post}] = l^* P/\omega^*$ when $C_L(\cdot)$ is logarithmic,

but also reduces to the derived macroscopic utility for a linear $C_L(\cdot)$ (when $k = 1$, see Eqs.(S-13) and (S-14)). Furthermore, as required for self-consistency, the Lagrange multiplier ξ that leads to the policy $\pi^*(\tau_L) \propto \exp(\beta(C_L(\tau_L) - \xi\tau_L))$ is the "shadow price" $\xi = \rho^* = \frac{RI+\mathbb{E}_{\pi^*}[C_L(\tau_L)]}{P+\mathbb{E}_{\pi^*}[\tau_L]}$, which is the average reward rate. If we had enforced the full budget constraint via a Lagrange multiplier, the same average reward rate would have been the shadow price for this too, i.e., the extra (macroscopic) utility arising from relaxing the total budget T, (i.e. taking an extra second total time for work and/or leisure).

References

1. Puterman ML (2005) Markov Decision Processes: Discrete Stochastic Dynamic Programming (Wiley Series in Probability and Statistics). Wiley-Blackwell, 684 pp.

2. Niv Y, Daw ND, Joel D, Dayan P (2007) Tonic dopamine: opportunity costs and the control of response vigor. Psychopharmacology 191: 507–20.

3. Cools R, Nakamura K, Daw ND (2011) Serotonin and dopamine: unifying affective, activational, and decision functions. Neuropsychopharmacology : official publication of the American College of Neuropsychopharmacology 36: 98–113.

4. Dayan P (2012) Instrumental vigour in punishment and reward. Eur J Neurosci 35: 1152–1168.

5. Niyogi RK, Breton YA, Solomon RB, Conover K, Shizgal P, et al. (2013) Optimal indolence: a normative microscopic approach to work and leisure. Journal of The Royal Society Interface 11: 20130969–20130969.

6. Kacelnik A, Marsh B (2002) Cost can increase preference in starlings. Animal Behaviour 63: 245–250.

7. Daw ND, Touretzky DS (2002) Long-term reward prediction in TD models of the dopamine system. Neural Computation 14: 2567–83.

8. Sutton R, Barto A (1998) Reinforcement learning: An introduction, volume 28. Cambridge University Press.
